# Resilience-based interventions in the public sector workplace: a systematic review

**DOI:** 10.1186/s12889-024-21177-2

**Published:** 2025-01-28

**Authors:** Malin H. L. Hollaar, Bram Kemmere, Paul L. Kocken, Semiha Denktaş

**Affiliations:** Erasmus School of Social and Behavioural Sciences, Erasmus University Rotterdam, Burgemeester Oudlaan 50, 3062 PA Rotterdam, The Netherlands

**Keywords:** Psychological resilience, Mental health, Health promotion, Occupational health, Intervention studies

## Abstract

**Background:**

Previous studies have advocated the benefits of resilience-based interventions for creating a healthy and sustainable workforce. However, resilience is defined and measured in diverse ways. Therefore, the aim of this systematic review is (1) to identify how resilience is defined within different workplace interventions, translated into intervention content, and measured in these interventions; and (2) to synthesize the effectiveness of these interventions.

**Methods:**

A systematic literature search was conducted and included articles from 2013 – 2023. Twenty-four studies met the inclusion criteria, covering a total of 26 unique interventions. Definitions were categorized as: resilience as a trait, process, or outcome. Cohen’s D was calculated to depict the effect sizes within the intervention groups from pre-test to post-test and, when possible, from pre-test to 3-month follow-up.

**Results:**

Included studies applied a wide range of definitions; most definitions fitted within the trait-orientation, conceptualizing resilience as an individual characteristic or ability, or the process-orientation, conceptualizing resilience as a dynamic process. No studies solely used the outcome-orientation, but some did combine elements of all three orientations. Various definitions, measures and intervention strategies were applied, however, almost half of the studies (46%) showed inconsistencies within these choices. Furthermore, findings show that most resilience-based interventions in the workplace have a positive impact. While educational workshops with a higher frequency and duration had medium to large effects, solely digital interventions had small effects, changing to small to medium when combined with non-digital elements.

**Conclusions:**

Findings suggest that resilience-based can benefit employees by enhancing their psychological well-being. This, in turn, can lead to improved work-related outcomes such as productivity, thereby offering advantages to employers as well. This underscores the growing recognition that resilience should be viewed as a shared responsibility between the individual and the organization. Further advancement in the field of resilience-based interventions in the workplace calls for future research to focus on maintaining consistency when choosing a definition of resilience, developing intervention content, and choosing an outcome measure.

**Preregistration:**

The search protocol was preregistered in the Open Science Framework, see Hollaar et al. (2023). 10.17605/OSF.IO/UKYF7.

**Supplementary Information:**

The online version contains supplementary material available at 10.1186/s12889-024-21177-2.

## Background

Since the last two decades, researchers and HR professionals have become increasingly interested in the effect of psychological wellbeing on work-related outcomes such as productivity [1–3]. Training employees how to manage stress and cope with workplace stressors can make them healthier and more engaged with their work. This is particularly important for public sector employees, who often operate in high-pressure environments such as health care and public safety. The public sector is frequently characterized by performance-based reforms, budgetary constraints, bureaucratic demands and intensified workloads, all of which contribute to higher stress levels [4–6]. Implementing resilience-based interventions could therefore be seen as a strategy to sustain a healthier and more resilient workforce within the public sector [7].

## Defining Resilience

Defining ‘resilience’ is a challenge [8–11]. Often resilience can be defined as the ability to bounce back from stress or the process of positively adapting to adversities [12, 13]. However, a brief examination of the existing resilience literature shows a proliferation of definitions [11, 14]. Previous studies tried to provide clarity by distinguishing three definitional categories of resilience. Most definitions either conceptualize resilience as a trait, as an outcome, or as a process [15]. In the first category, resilience is seen as a personal trait that protects someone against the potential impact of adverse events. It is considered a personal, intrinsic characteristic [15, 16], but some researchers in the trait orientation define it as an ability. This would imply that resilience can be trained [16, 17]. Both views belong to the trait orientation since both see resilience as (1) an intrinsic quality; and as (2) independent of adversity exposure.

However, this trait view has faced criticism for its limitations, such as the observation that even highly resilient individuals may struggle under significant stress and the observed influences of external factors [10, 18–20]. These critiques have led to the development of other perspectives on resilience. Such as a second category, in which resilience is seen as an adaptive outcome, defining it as the maintenance or restoration of mental health after exposure to a significant adversity. The focus is on the present exposure to an adversity and the corresponding coping process. This success of the coping process is measured in terms of mental health outcomes such as personal growth or wellbeing [15]. These mental health outcomes can be modified by so-called resilience factors, which can consist of internal resources (e.g. self-efficacy and coping abilities) and external assets (e.g. presence of social support) [9, 15, 21, 22]. In the last category, resilience is seen as a process, and is often defined as the dynamic process of adaptation itself, which is characterised by the utilization of internal resources and external assets. These protective factors, i.e. internal resources and external assets, facilitate the adaptation to the adversity, leading to better outcomes [11, 20]. The process orientation considers the phases before, during and after adversity exposure when investigating the protective factors and the adaptation to the adversity [16]. Post-adversity, resilient individuals either show stable mental health, or only a temporary disturbance, followed by a relative quick recovery, or even growth [15]. Lastly, the process of resilience can vary in different contexts and domains [16, 23].

In summary, within the trait orientation resilience is an individual characteristic and independent of adversity exposure, while in both the process- and outcome orientation it depends on adversity exposure. The outcome orientation focuses on the result (e.g. stable or increased wellbeing), while the process orientation emphasizes the journey rather than solely the outcome [15, 16]. In both orientations, the influence of external assets on resilience opens possibilities for interventions aimed at improving resilience and mental health [20, 24].

### Research Aim

As workloads get heavier and burn-out rates are rising, more employers seem interested in resilience-based interventions to sustain a healthy and resilient workforce [4, 25]. The popularity of resilience-based interventions in the workplace is increasing, yet research into the topic is hampered by the heterogeneity and inconsistencies displayed by the literature on resilience [26]. Previous reviews included interventions up until 2014 and have analysed whether resilience-based interventions can indeed improve resilience in the workplace. However, these previous reviews were affected by the varying quality of study designs and inconsistencies in definitions, intervention content and assessments of resilience [4, 27]. Ideally, the selection of a specific definition of resilience establishes theoretical boundaries, which shapes both the intervention content and the chosen measurement instruments. In the absence of consensus on the definition of resilience, studies have exhibited diversity in intervention approaches and outcome measurement. As a result, studies produced a spectrum of outcomes said to measure resilience. Discrepancies or inconsistencies in resilience definitions, intervention content and measurement will create a jingle-jangle problem. This means that either there is one label being used for different meanings (jingle) or that different labels are used for the same, single meaning (jangle). A jingle-jangle problem will therefore hinder the comparison of research findings on the topic of resilience [9, 28]. What remains unclear is whether the jingle-jangle issues persist in more recent research.

Resilience-based interventions in the workplace, thus, show variability in theoretical backgrounds, intervention approaches, and choice of measurement instruments. This variability hampers the ability to compare and determine the effectiveness of these interventions. A comprehensive overview can help to elucidate the conceptual underpinnings of resilience-based interventions and their practical applications in the workplace. Whereas previous reviews have noted inconsistencies, this study sets out to document the current state of the variability by separating different categories of resilience (i.e., trait, outcome and process), evaluating whether conceptual definitions are in line with the operational definitions (i.e., measurement instruments) and the intervention content (i.e., individual-focused, organizational-focused, or combined), and to investigate which interventions are effective. Additionally, since the topic of resilience has increased in popularity, an expanded body of research on resilience-based interventions now allows for deeper insights into whether the issues identified in older research persist, have worsened, or have improved. The aim of this systematic review is twofold: (1) to identify how resilience is defined within different workplace interventions, translated into intervention content, and measured in these interventions; (2) to synthesize the effectiveness of resilience-based interventions in the workplace.

## Method

### Search Procedure and Strategy

A systematic literature search was performed according to the Preferred Reporting for Systematic Reviews and Meta-analysis (PRISMA) [29]. Our completed PRISMA 2020 Checklist was added in Appendix IV [30]. The search protocol was preregistered in the Open Science Framework, see [31]. The search was conducted in PubMed, PsycINFO (via OVID), Business Source Premier, Scopus, and Web of Science. Articles were included up to 6th of April 2023. Variations of the following keywords were combined: “resilience”, “intervention”, “employment” and “experimental design” (see Table 1). These keywords were added to the search query together with synonyms or MeSH terms. The search queries for each database can be found in Appendix I.

**Table 1:** Search terms

Resilience	Interventions	Employed	Experimental design
Psychological resilience	Intervention*	Employment	Randomized controlled trial*
Resilien*	Program*	Personnel	RCT
Hardiness	Training*	Work*	Empirical study
Distress tolerance	Promotion*	Employees	Quasi-experimental*
	Enhancement*		Observational study
	Education		

In case any literature reviews were encountered with the search queries, they were not excluded immediately. We applied the snowballing technique and looked through their reference lists, searching for potentially relevant studies to include. Last consensus about the inclusion of studies through snowballing was reached on the 26th of May 2023.

## Eligibility Criteria

A predetermined list of eligibility criteria was used to assess articles for inclusion. We opted to solely include the public sector, thus, excluding studies that took place in the private sector due to the variation in work activities, occupational structures, and possible sources of stress [32]. While we acknowledge the debates on whether occupational stress is different between these sectors, focusing solely on the public sector allowed us to maintain more coherence in our sample. For similar reasons, studies that took place in the military were also excluded, due to the differences in their responsibilities and sources of stress. Furthermore, to be included the theoretical background of the article should contain a definition or theory of resilience. Additionally, since we focus on psychological resilience, the research should concern human behaviour. Research looking into resilience beyond human decision making, such as marine biology and computer sciences, were excluded. The full list of eligibility criteria can be found in Table 2.

**Table 2:** Eligibility Criteria

	**Inclusion**	**Exclusion**
Type of Article	Full-text, peer-reviewed, English, published between 2013–2023	Other languages, published before 2013
Types of Studies	Intervention studies; randomized-controlled trial (RCT), quasi-experimental, observational	Non-empirical studies
Study Design	At least one pre-test and one post-test measurement, presence of at least one comparison group	Only a post-test measurement, no comparison group
Types of Participants	Employed adults, working in a public sector organization	Students, retirees, unemployed, employees in the military, private sector employees^1^
Types of Interventions	Resilience-based interventions: explicit mention of resilience as a primary aim, method, or outcome. Article contains description of intervention content	No intervention description. Resilience was merely a ‘by-catch’ and not the main aim
Theoretical Background	Description of resilience (definition, theory). Research concerning human behaviour (such as sociology, psychology, health sciences)	Only mentions resilience without theoretical background or definition. Research beyond human decision making and functioning (such as resilience in computer systems and ecological systems)

^1^In case the sector was unspecified, or the study took place in both the private and public sector, the article was considered for inclusion

## Study Selection and Search Results

During selection, the Rayyan.ai was utilized to support the process [33]. The application identified duplicates, which were cross verified by two reviewers (MH and BK) before removal. Titles and abstracts were independently screened by both reviewers based on predefined eligibility criteria, and any disparities were resolved through discussion with a third author (PK). Inter-rater reliability during the screening stage reached a 94.74% agreement.

In the subsequent round, the first reviewer (MH) assessed article introductions and methods, excluding studies lacking definitions of resilience and those with an inappropriate study design. Any doubts were discussed together with the second and third author (BK and PK). A final selection of 31 studies was agreed upon.

**Fig. 1:**
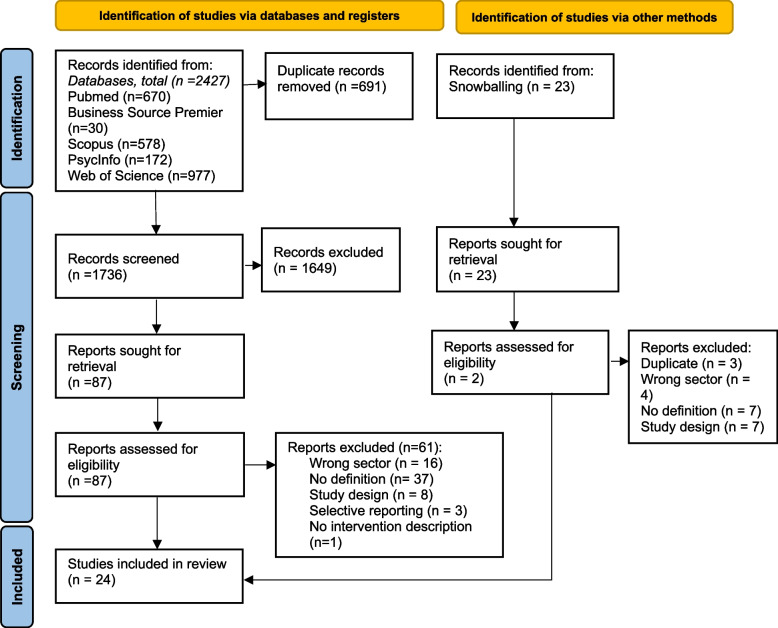
PRISMA Flowchart of search results and selection, adapted from [30]

At the full-text level, data extraction by two reviewers (MH and BK) led to the additional exclusion of seven studies. Four studies were encountered that, at full-text level, did not meet the eligibility criteria. An additional three studies were found to be of critical risk of bias, due to selective reporting. Agreement between three reviewers (MH, BK, and PK) was reached to exclude these seven studies, leaving a final selection of 24 studies. The PRISMA flow chart (see Fig. 1) depicts the selection process.

## Data extraction and quality appraisal

Data was extracted by two reviewers (MH and BK). The data extracted concerned demographic characteristics of the study population, study design, theoretical background, study outcomes and a summary of the intervention content and its duration and frequency. The interventions’ content was synthesized by MH according to their theoretical framework, intervention strategy, and whether they were primarily aimed at factors on the individual level (internal) or outside of the individual level (external). With intervention strategy, we mean the broad approaches and techniques that were used to achieve the desired outcomes or behaviours [34, 35]. MH and BK categorized definitions and measurement instruments based on the resilience definitions: trait, process, or outcome. No formal intercoder reliability was calculated. MH and BK held regular meetings to discuss data extraction and ensure consistency. Later, MH maintained regular feedback sessions and discussions with PK to ensure accuracy of intervention strategies and internal and external factors.

The risk of bias of the included studies was assessed independently by the same reviewers (MH and BK) using the Risk of Bias in Non-randomized Studies of Interventions (ROBINS-I). The following domains were critically assessed: bias due to confounding, bias in selection of participants, bias in classification of interventions, bias due to deviations from intended interventions, bias due to missing data, bias in measurement of outcomes, and bias in selection of the reported result [36, 37]. Any disagreements between the two reviewers were resolved by consulting a third review author (PK). Last consensus about risk of bias was reached on the 5th of October 2023.

## Calculation of effect sizes

Effect sizes were calculated to gain insight into the effectiveness of the resilience-based interventions. We anticipated we would find two types of resilience-based interventions: resilience-directed and resilience-mediated intervention. We will divide the effect size calculations accordingly. Resilience-directed interventions do not primarily use resilience as a mean, but target participants’ resilience using other methods and always measure resilience as an outcome. These interventions use numerous ways of targeting resilience, like mindfulness or biofeedback training. For resilience-directed interventions, effect sizes were calculated for the primary resilience measure. Resilience-mediated interventions use the concept of resilience as a mean, for example by teaching skills related to resilience. These interventions do not necessarily measure resilience but measure the effects of resilience training on another type of outcome. In this case, we calculated the effect sizes of the measures they appointed as primary outcomes.

Cohen’s D was calculated to depict the effect sizes within the intervention groups, between pre-test and post-test measurement and, when possible, between pre-test and 3-month follow-up measurement. We used the following formula: (M_1_-M_2_)/SD_12_ with SD_12_ as the pooled standard deviation. To interpret the results, the revised rules of thumb for effect sizes interpretation were used. When *d* = 0.01, it is considered to be very small. A *d* = 0.2 is considered to be small, *d* = 0.5 is considered to be medium, and *d* = 0.8 is considered to be large [38, 39]. In theory, Cohen’s D can range from negative infinity to positive infinity. However, extremely large values (e.g. d ≥ 4) are rarely found and often indicative of methodological issues, such as outliers or measurement errors.

## Results

### Aim 1: Defining, Translating, and Measuring Resilience in Workplace Interventions

#### Resilience definitions and measurement

We included 24 studies that each investigated one unique resilience-based intervention, except for Hsieh et al. [49] and Mistretta et al. [77] who each studied two resilience-based interventions in their respective studies. Therefore, there are 26 interventions included in this review. Within the included studies, we found definitions that fitted within the trait orientation and the process orientation. There were no definitions that solely fitted within the outcome orientation; however, we did encounter definitions that contained a combination of elements of the trait orientation, process orientation, and/or outcome orientation (see Table 3).

**Table 3:** Resilience Definitions and Operationalizations

Study	Definition and corresponding category	Intervention Name^a^	Intervention: Theory	Intervention: Strategy	Intervention: Duration and Frequency	Internal or external factors^b^	Public Sector Area	Measurement Instrument and corresponding category^c^
Chermack, 2017 [40]	**Self-resilience**: the capacity to move on in a positive way from negative, traumatic or stressful experiences. The concept of resilience is typically subdivided into several essential traits: purpose, perseverance, equanimity, self-reliance and existential aloneness. **Trait**	Scenario Planning	Theory of Scenario Planning [41]	**Educational workshop, group-based:** learning based on real-life scenarios and their possible solutions	**Total:** 5 sessions	Internal, external	NR	RS: trait
Chitra and Karunanidhi, 2021 [78]	**Resilience:** in policing context, resilience is both psychological and physiological flexibility in the face of adversity, self-awareness, and control over one’s physiological stress responses to threat and recovery from exposures beyond one’s control. **Trait**	Resilience Training	Protective Model of Resilience [42], Transactional Model of Stress and Coping [43], principles of Positive Psychology	**Educational workshop, group-based:** learning skills related to resilience through lectures, modelling, role-playing, reflection and relaxation techniques	**Total**: 20 sessions in 2 months, **per week:** 3 1.5-h sessions	Internal	Public Safety	CD-RISC: trait
Fernandes, 2019 [44]	**Teacher resilience:** resilience can be understood as a multidimensional psychological construct, which is socially constructed. It refers to an adaptive functioning and development in situations where challenge and adversity are present, entailing purpose and meaningful actions. **Trait, process and outcome**	Positive Education	Education for Wellbeing	**Educational workshop, group-based:** learning skills related to teacher resilience through lectures, practices, and case-based discussions	**Total:** 9 sessions, **per week:** 1 2-h session	Internal	Education	Teacher Resilience Scale: trait
Franco, 2021 [45]	**Resilience:** the ability to “bounce back” after encountering stressors, to respond to stress in a healthy way and adapt in the face of adversity, setbacks, or trauma. Ability to disconnect from work in order to recharge (resilience decompression) and the ability to feel engaged and rewarded by work (resilience activation). **Trait**	Self-Compassion for Healthcare Communities	NR	**Educational workshop, group-based:** learning self-compassion through writing activities, practices, group exercises, and discussions	**Total:** 1-day workshop, 6 h	Internal	Healthcare	SRS: trait
Grabbe, 2019 [46]	**Resilient Zone**: an internal state of balance where we are at our best, able to learn, solve problems, and work effectively with others. In the Resilient Zone or “OK” Zone, an individual is emotionally stable and capable of adapting to challenges or learn from them. **Trait**	Community Resilience Model	Somatic Experiencing	**Educational workshop, group-based:** learning sensory awareness skills through lectures, engagement, discussion, demonstration, and participation. **Digital intervention:** access to the CRM 'ichill' app	**Total:** one 3-h session	Internal	Healthcare	CD-RISC10: trait
Hasani, 2022 [47]	**Resilience**: an ability to adapt to adversity adaptability and as a form of strength or flexibility in the face of a stressor. **Trait**	Resilience Skill Training	NR	**Educational workshop, group-based:** learning skills related to resilience	**Total:** 8 sessions, **per week:** 2 45-min sessions	Internal	Healthcare	n.a
Henshall, 2023 [48]	**Resilience**: an individual’s ability to “adjust to adversity, maintain equilibrium, retain some sense of control over their environment, and continue to move on in a positive manner”. **Trait**	REsOluTioN	NR	**Digital intervention:** learning skills related to resilience through online synchronous and asynchronous learning and mentor support	**Total**: 4 weeks, **per week:** 1 × 120 min group session, 1 × 30-min independent preparatory learning, and 2 × small group mentoring sessions 30–60 min	Internal	Healthcare	BRS: trait
Hsieh, 2020 [49]	**Resilience**: the process of adapting well in the face of adversity, trauma, tragedy, threats, stress, serious health problems, or workplace conflict—it means ‘bouncing back’ from di fficult experiences. **Process**	Biofeedback training	NR	**Educational workshop:** incorporating aspects of real-time respiratory sinus arrhythmia (RSA)-biofeedback and shorter meditation practices	**Total:** six sessions, **per week:** one 1-h session	Internal	Healthcare	RSA: process
		Smartphone-based biofeedback training	NR	**Digital intervention:** smartphone app with guided meditation practices and real-time biofeedback	**Total:** six sessions, **per week:** 1 session	Internal	Healthcare	RSA: process
Im, 2016 [50]	**Ego-resilience**: one’s ability to adapt appropriately to the presence of significant adversity or stress and bounce back from a stressful or adverse situation as a strengthened and more resourceful person with flexibility and positive emotionality. **Trait**	Huddling Programme	NR	**Team building activities and educational workshops, group-based:** learning several psychosocial skills and encouraging social cohesion and support through social activities and trainings. **Digital intervention:** a smartphone app to support participants unable to attend to offline gatherings	**Huddling Programme**: 1 full-day, **After-Work Huddling Programme:** 3 sessions, 1 × per 2 weeks, **SNS Huddling Programme:** smartphone app, accessible during 2 months	Internal, external	Healthcare	ER-89: trait
Janzarik, 2022 [51]	**Resilience**: a multidimensional and dynamic process of positive adaptation to stress, which involves a complex interaction between individual traits and the environment the individual is living in. The result of this process is characterized by the maintenance or quick recovery of mental health during or after stress. **Process**	The New Growth	Psychodynamic model focused on stress development	**Educational workshop, group-based:** learning stress-coping skills through CBT, psychodynamic psychotherapy, mindfulness and imagination exercises	**Total:** 8 sessions, **per week:** 1 2-h session	Internal	Healthcare	BRS, CD-RISC: trait Resilience-related constructs: subjective wellbeing (WHO-5), general self-efficacy (SWE), self-esteem (RSES), satisfaction with life (SWLS), emotion regulation skills (ERSQ-27), perceived stress (PSS)
Joyce, 2019 [52]	**Resilience:** there are two inherent constructs of resilience: (1) successful adaptation to stressful life events and circumstances and (2) bounce-back resilience. Resilience can be viewed as a dynamic process reflecting a person’s ability to adapt, manage, and recover effectively from stressful experiences and adverse circumstances. **1) Outcome; 2) Trait and Process**	Resilience@Work [RAW] Mindfulness Program	Principles of mindfulness and ACT	**Digital intervention**: iPad application focused on learning self-compassion and acceptance skills through mindfulness and ACT	**Total:** 6 sessions. **Frequency:** min. 3-day break between sessions, and max. 1 week. **Each session:** 25 min	Internal	Public Safety	CD-RISC-10, BRS: trait Resilience resources: optimism (life orientation test-revised), coping (the brief-coping orientation to problems experienced), sense of purpose (life engagement test)
Mache, 2015 [53]	**Resilience:** the ability of an individual to withstand adversity and is often seen as a form of self-recovery with positive emotional and cognitive outcomes, which in turn has an important role in realizing greater adaptability and life satisfaction. **Trait**	Multicomponent Psychosocial Skills Training	Aspects from CBT and solution-focused counseling	**Educational workshop, group-based:** learning coping strategies, and solution and goals for the future, and facilitating social support through principles of CBT and solution-focused group work	**Total:** 12 weeks, **per week:** one 2-h session	Internal	Healthcare	BRCS: trait
Mahaffey, 2021 [54]	**Resilience**: in this context resilience is conceptualized as the adaptive use of coping skills such as utilizing social supports, engaging in self-care activities, and improving health behaviors. **Process**	Disaster Worker Resiliency Training Program (DWRT)	NR	**Educational workshop, group-based**: learning about signs and symptoms of disaster work-related stress, help-seeking behavior and strategies, and stress reducation and coping strategies	**Total:** one 4-h session	Internal	Public Safety	n.a
Mao, 2021 [55]	**Resilience**: the individual's ability to maintain a stable equilibrium and bounce back from adversity.** Trait**	Emotional Intelligence Training	Four-branch Model of Emotional Intelligence [56]	**Educational workshop, group-based:** lectures about emotional intelligence and case-based group discussions	**Lectures** 2 × per week for one month, **Workshops** 44 60–90 min session, 1 per week	Internal	Healthcare	CD-RISC: trait
Marais, 2016 [57]	**Resilience:** an adaptive quality in the presence of adversity, contributing to independent functioning and well-being. A dynamic process in which the individual positively adapts to adversity or risk. After a stressful event, the resilient individual has the capacity to rebound and attain a healthy outcome. **Trait and process**	Sensory Stimulation Therapy	NR	**Environmental modification:** implementation of a sensory stimulation room in the hospital	**Total:** available during 2 months	Internal, external	Healthcare	RS: trait
Mealer, 2014 [58]	**Resilience** is a psychological characteristic that has been defined as a trait or capacity depending on the underlying theory adopted. Resilience can be learned and has also been recognized as one of the most important factors in successful adaptation following exposure to a traumatic event. Specific factors that can promote resiliency include positive support systems, attention to physical well-being, and development of active coping skills. **Trait**	Multimodal Resilience Training Program	Principles of MBSR and CBT, Pennebaker’s expressive writing framework	**Educational workshop:** guided mindfulness sessions, written exposure therapy, practice of stress reduction techniques, aerobic exercises, and CBT sessions	**Total:** 12 weeks. **Mindfulness**: two 2-h guided sessions, 1 per week. **Written exposure**: a 4-h introduction. Each week, a 30-min session. **Stress reduction techniques**: each week, min 3 × 15 min. **Aerobic exercise**: 30 to 45 min at least 3 days. **Cognitive behavioral therapy:** each session 30 to 60 min	Internal	Healthcare	CD-RISC: trait
Mistretta, 2018 [77]	**Resilience:** adaptive responses to stress. Individuals or communities that exhibit resilient responses to stressors remain committed to values and goals despite experiencing adversity. The strengths within individuals and community members to persevere and recover through environmental, physical, or emotional stress. **Trait**	Mindfulness based resilience training	Aspects of ACT and MBRT	**Educational workshop, group-based:** learning about skills related to mindfulness and resilience, practicing meditation and mindful movement	**Total:** 6 weeks, **per week:** one 2-h session	Internal	Healthcare	n.a
		Smartphone resiliency training		**Digital intervention:** smartphone app aimed at increasing awareness about sleep, wellbeing and emotions, while identifying potential targets of change	**Total:** 7–10 days	Internal	Healthcare	n.a
Pidgeon, 2014 [59]	**Resilience:** the competence to cope and adapt in the face of adversity and to bounce back when stressors become overwhelming. **Trait**	Mindfulness with Metta Training Programme (MMTP)	Aspects of mindfulness practice with loving-kindness meditation, and cognitive therapy	**Educational workshop, group-based:** residential retreat focused on mindfulness and self-compassion through CBT, periods of silence, and practice of mindfulness and metta skills	**Total:** one 2.5-day retreat, two 4-h booster sessions	Internal	NR	RS-14: trait
Slatyer, 2018 [60]	**Resilience:** the process of adapting well in the face of adversity, trauma, tragedy, threats or significant sources of stress. It is generally accepted that resilience is a multidimensional construct made up of both trait-based aspects and skills that can be learned. **Process and trait**	MSCR	Compassion Fatigue Prevention and Resiliency: Fitness for the Frontline [61], mindfulness concepts and practices adapted from Segal et al. [62]	**Educational workshop, group-based:** learning about compassion fatigue through lectures and learning skills related to resiliency through mindfulness sessions	**Total:** 4 weeks. One full-day educational workshop and **per week:** 1 follow-up sessions of 105-min	Internal	Healthcare	CD-RISC: trait
Spilg, 2022 [63]	**Resilience**: the personal qualities that enable individuals to thrive when facing adversity. **Trait**	Stress Management and Resilience Training (SMART)	Principles of AIT	**Educational workshop, group-based:** learning self-care practices. **Digital intervention:** optional online program focused on the skills taught in the workshop	**Total:** One 2-h workshop, **optional**: 24-week online program	Internal	Healthcare	CD-RISC: trait
Tonkin, 2018 [64]	**Resilience**: a combination of assets and resources within the individual and their environment that facilitate the individual's capacity to adapt in the face of adversity. This definition- acknowledges psychological mechanisms and contextual factors that contribute to resilience. Resilience falls on a continuum, can be exhibited at differing degrees across multiple life domains, and should be viewed as context dependent. **Process** **Employee resilience:** employee capability, facilitated and supported by the organization, to utilise resources to continually adapt and flourish at work, even when faced with challenging circumstances	Wellbeing Game	The Five Ways to Wellbeing [65]	**Digital intervention**: online game with competitional elements in which participants document the time spent on wellbeing activities	**Total:** available for 4 weeks	Internal, external	Government, Education	EmpRes, CD-RISC10: trait
Van Der Meulen, 2018 [66]	**Psychological resilience:** as being able to perform under stress and not be affected by its potentially harmful consequences. It is a malleable characteristic of individuals and therefore the concept is central to stress management interventions. **Trait**	Mental Strength Training	Three domains of Mental Toughness (challenge, control and confidence)	**Educational workshop, group-based:** learning about mental strength the corresponding skills	**Total:** 3 days consecutive, **each sessions:** 8 h	Internal	Public Safety	MTQ-48, RS-nl: trait
Weber, 2019 [67]^1^		Kelaa Mental Resilience App	Insights from psychology, sleep medicine, and neuroscience	**Digital intervention:** smartphone app with psychoeducational content about emotions and mindfulness	**Total:** self-directed, possibility of daily for a total of 28 nights	Internal	Public and private	RS-13: trait
Wild, 2020 [68]	**Resilience:** the capacity to maintain wellbeing in response to adversity or stress. **Trait**	Resilience Intervention	Principles of mindfulness and stress management	**Digital intervention:** smartphone app with psychoeducational content about emotions and mindfulness	**Total:** 6 weeks, **per week:** one 2.5-h session	Internal	Public Safety	CD-RISC: trait

^a^Not all studies described the name of the intervention, in this case we wrote down the term they used to describe the intervention (e.g. Resilience Training or Resilience Skill Training)

^b^Internal: the intervention is focused on factors within the individual level, such as coping and confidence; External: the intervention is focused on factors outside of the individual. It is focused on factors within other socioecological levels, such as social support and organizational change

^c^More details about the resilience questionnaires and their theoretical backgrounds can be found in Appendix II

^1^describes various definitions of resilience but does not clearly state which one they used within their study

*ACT* Acceptance-Commitment Therapy, *AIT* Attention and Interpretation Therapy, *CBT* Cognitive Behavioral Therapy, *MBRT* Mindfulness-Based Resilience Training, *MBSR* mindfulness-based stress reduction, *n.a*. not applicable, *NR* not reported

The majority of the studies fitted within the trait orientation. A total of fifteen studies viewed resilience as a trait, for example Mao et al. (2021, p.2) who defined resilience as ‘*the individual’s ability to maintain a stable equilibrium and bounce back from adversity’*. Most of these studies either used the Connor-Davidson Resilience Scale (CD-RISC) [69], the CD-RISC10 [70], or the Resilience Scale (RS) [71, 72], or a shorter, adapted version like the RS-13 or RS-14 [73–75]. Other studies used a unique scale, such as the Brief Resilience Scale (BRS) [13]. The theoretical background of these measures fit within the trait-orientation and are thus consistent with the chosen definitions. Appendix II contains a full overview of all included resilience scales and their theoretical backgrounds.

There were four studies that fitted in the process orientation, describing resilience as a dynamic process in which an individual adapts successfully from an adversity. For example, Mahaffey et al. (2021, p.10) described resilience as ‘*the adaptive use of coping skills such as utilizing social supports, engaging in self-care activities, and improving health behaviours.’.*

Lastly, there were four studies with definitions that combined trait-oriented elements with either outcome-related or process-related elements. Of those four studies, two combined elements from the trait-orientation and process-orientation [57, 60]. These studies describe resilience as an adaptive quality, while highlighting its dynamic nature. The other two extend this understanding by adding outcome-oriented elements. For example, Joyce et al. [52] identified two separate meanings of resilience, the first being successful adaptation after a stressful situation and the second being resilience as a dynamic process that reflects one’s ability to adapt and recover. These two meanings highlight elements from the trait-orientation, as well as the process- and outcome-orientation.

All studies in the process orientation and all studies that combined elements from multiple orientations adopted a distinctive approach to measure resilience, either with one measure or with a combination of measures. For example, Hsieh et al. [49] defined resilience as a process and was the only study that used the Resilience Scale for Adults (RSA) [76]. There were three studies that used a combination of multiple scales to measure resilience [51, 52, 64]. Lastly, we found three studies that did not measure resilience. These evaluated the effects of a resilience-mediated intervention on a different outcome, such as occupational stress [47, 54, 77].

### Intervention content

Of the 26 included interventions, most intervention strategies were concerned with group-based educational workshops (see Table 3 for an overview of intervention approaches, resilience definitions and measurement scales). Most of these educational workshops were concerned with teaching their own selection of skills related to resilience, such as emotion management, interpersonal skills and self-reflection [40, 44, 47, 50, 78], a selection of skills related to mindfulness [58–60, 77], or skills related to stress and coping [51, 53, 54]. Some interventions focus on a specific psychosocial skill, like self-compassion, emotional intelligence or mental strength [45, 55, 66]. Other educational workshops were about self-care practices [63], sensory awareness [46], biofeedback and meditation [49], or focused on scenario planning [40]. For more details about the educational content, see Table 3. Three of these group-based interventions offered the additional opportunity to continue learning and training with an online app or website. Whereas seven interventions were fully smartphone- or app-based. Three of these digital interventions contain psychoeducational content aimed at emotions or self-compassion by means of mindfulness [52, 67, 68]. Two are aimed at well-being, one through game-based elements [64] and one through goal setting aimed at awareness, sleep and emotions [77]. One has psychoeducational content about skills related to resilience, such as emotional intelligence and reflective thinking [48]. Regarding factors directed at the individual (internal) and factors directed at the environment (external), most interventions mainly targeted internal factors, with four interventions incorporating additional external factors, such as social support or organizational (re)structuring. In terms of duration and frequency, the interventions varied a lot in intensity; the shortest being one 2-h workshop [63] and longest consisting of 52 sessions [55]. Most studies (14 total) were conducted in the healthcare setting and the public safety setting (5 total). The educational sector was researched by two studies. Lastly, three studies did not specify which sector they investigated.

Focusing on potential inconsistencies, further analysis showed that a small majority of the included studies were consistent with their choice of definition, intervention content, and measurement instrument. However, eleven studies did show inconsistencies, six of which showed inconsistencies in their choice of definitions and measurement scales (see Table 3). They chose process definitions, or trait and process combined, but only used a trait-oriented measurement scale. However, two of these studies seem to do this on purpose to strengthen their arguments in favour of another measurement instrument [44, 64]. For example, Tonkin et al. (2018) used the CD-RISC [69] and the Employee Resilience Scale (EmpRes) [79] to measure personal resilience and employee resilience. In their analysis they showed that these two constructs are indeed separate constructs and, thus, should be measured accordingly. Lastly, the other five studies showed inconsistencies between intervention content and measurement. For example, the study of Pidgeon et al. (2014) used the RS-14 [73], measuring five resilience characteristics, whereas their mindfulness program did not explicitly address any of these characteristics.

### Aim 2: Effectiveness of Resilience-Based Interventions in the Workplace

#### Study Design and Risk of Bias

For evaluating their respective interventions, the 24 included studies varied in targeted population, design, and outcome measures. Table 4 contains a summary of the study designs and population characteristics.

**Table 4:** Study Design and Participant Characteristics

Study	Method	Time Points Measured	Primary Outcomes	Intervention (n =)	Control (n =)	Country	Population	% Female	Ethnicity
Chermack, 2017 [40]	QE	Pre, post	Resilience	48	No Intervention (44)	Western USA	NR	NR	NR
Chitra and Karunanidhi, 2021 [78]	RCT	Pre, post, 2-month follow-up	Occupational stress, Resilience, Job Satisfaction, Psychological Wellbeing	33	No intervention (30)	South of India	Female police officers	100	NR
Fernandes, 2019 [44]	QE	Pre, post	Teacher’s Resilience, Motivation, Self-efficacy, Commitment to the Profession, School Support, Positive and Negative Experiences, Work meaning, Wellbeing	Group 1: 17, Group 2: 18	Waitlist Control (24)	Portugal	Teachers (primary, 2nd, 3rd cycle and secondary education)	78	NR
Franco, 2021 [45]	QE	Pre, post, 3-month follow-up	Self-compassion, Mindfulness, Compassion, Resilience, Job Engagement, Professional Quality of Life, Depression, Anxiety and Stress	22	No Intervention (26)	USA	Pediatric nurses	90	Latino: 10% Black: 4% White: 85%
Grabbe, 2019 [46]	RCT	Pre, post	Wellbeing, Resilience, Secondary Trauma Symptoms, Work-related Burnout, Somatic Symptoms	40	Nutrition Intervention (37)	USA	Nurses	95	NR
Hasani, 2022 [47]	RCT	Pre, post, 1 month follow-up	Nursing Stress	18	Metacognitive Training (18), no intervention (18)	Iran	Nurses	85	NR
Henshall, 2023 [48]	RCT, pilot	Pre, post^2^	Resilience, Psychological Wellbeing	56	Waitlist control (51)	United Kingdom	Nurses	89	89% White
Hsieh, 2020 [49]	QE with cluster random sampling	Pre, post	Depressive Symptoms, Resilience, Respiration Rate, Occupational Stress	BT: 49, SDBT: 47	No intervention (39)	Taiwan	Abused Pediatric Ward Nurses	88	NR
Im, 2016 [50]	RCT	Pre, post	Empowerment, Organizational Commitment, Ego-resilience	24	No intervention (25)	South Korea	Clinical nurses	82	NR
Janzarik, 2022 [51]	RCT	Pre, post, 6-and 9-month follow-up	Mental Health, Resilience	38	Waitlist control (34)	Germany	Nurses	92	NR
Joyce, 2019 [52]	Cluster RCT	Pre, post, 6-month follow-up	Resilience and Resilience Resources (Optimism, Coping, Sense of Purpose)	60	Healthy living program (83)	Australia	Firefighters	4	NR
Mache, 2015 [53]	RCT	Pre, 3-month follow-up	Perceived Distress, Resilience, Self-efficacy, Optimism, Job Satisfaction	42	No intervention (43)	Germany	Junior Physicians	62	NR
Mahaffey, 2021 [54]	RCT	Pre, 3-month follow-up	Healthy Lifestyle Behaviors, Perceived Stress, Depression, and PTSD symptoms	78	Waitlist control (89)	USA	Disaster workers: responder to Hurricane Sandy (professionally or voluntarily)	56	Caucasian 70% non-Hispanic 87%
Mao, 2021 [55]	RCT	Pre, post	Emotional Intelligence, Resilience, and Stress, Inpatient Experiences	53	Daily training from nursing department, no emotional intelligence content (50)	China	Medical and surgical ward nurses	NR	NR
Marais, 2016 [57]	QE	Pre, post	Resilience, Self-reported Items about SST	41	No intervention (23)	South Africa	Professionally trained theatre nurses	100	NR
Mealer, 2014 [58]	RCT	Pre, post	Resilience, PTSD Symptoms, Anxiety and Depression Symptoms, Burn-out Symptoms, Client and Patient Satisfaction	13	No intervention (14)	USA	Intensive Care nurses	89	White 100%
Mistretta, 2018 [77]	RCT	Pre, post, 3-month follow-up	Depression, Anxiety and Stress Symptoms, Wellbeing	MBRT: 22, SRT: 23	Waitlist control (15)	USA	Employees at Mayo Clinic, a large research hospital	87	NR
Pidgeon, 2014 [59]	RCT	Pre, post, 1-month follow-up, 4-month follow-up	Resilience, Mindfulness, Self-compassion	14	No intervention (16)	Australia	Human service professionals	91	NR
Slatyer, 2018 [60]	QE	Pre, post, 3-month follow-up, 6-month follow-up	Professional Quality of Life; Depression, Anxiety and Stress; Resilience; Self-efficacy; Self-compassion; Wellbeing	65	Waitlist control (26)	Australia	Nurses	67	Australian 60% Other 13% Not Specified 26%
Spilg, 2022 [63]	Exploratory RCT	Pre, 3-month follow-up, 6-month follow-up	Resilience, Subjective Happiness, Perceived Stress, Anxiety	16	No intervention (16)	Canada	Academic physicians	35	NR
Tonkin, 2018 [64]	QE	Pre, post	Employee Resilience, Personal Resilience, Self-related Health, Energy Levels	81	No intervention (52)	New Zealand	NR	85	NR
Van der Meulen, 2018 [66]	QE	Pre, 3-month follow-up, 9-month follow-up	Psychological Resilience, Mental Health Disturbances, Potentially Traumatic Events	138	No intervention (167)	Netherlands	Police officers	27	NR
Weber, 2019 [67]	Multi-center RCT	Pre, mid-intervention post and two-week follow-up	Stress, Subjective Wellbeing, Resilience, Social Community at Work, Sleeping Troubles, Physical Health Impairment, Work Productivity and Activity Impairment	322	No intervention (210)	Germany, England, Northern Ireland	Employees of public and private sector from six different European businesses	76	NR
Wild, 2020 [68]	RCT	Pre, post, 3-month follow-up	Wellbeing, Resilience, Self-efficacy, Problem-solving, Social Capital, Confidence in Managing Mental Health, and Number of Days off Work due to Illness	317	Psychoeducation (113)	United Kingdom	Emergency Workers	58	White/ European 94% Black/ Indian/ Asian/ Arab 6%

BT = Biofeedback Training, MBRT = Mindfulness-Based Resilience Training, NR = not reported, PTSD = post-traumatic stress disorder, QE = quasi-experimental, RCT = randomized controlled trial, SDBT = Smartphone-Delivered Biofeedback Training, SRT: Smartphone Resiliency Training, SST = Sensory Stimulation Therapy

^1^ COVID-19 pandemic began before 3-month follow-up

^2^ Whole data collection took place during the COVID-19 pandemic

Regarding study design, a majority (16 studies) conducted randomized controlled trials (RCT) and a minority (8 studies) was quasi-experimental (QE). The number and timing of data collection also varied. A majority (14 studies) collected data at two time points, mostly pre- and post-intervention. The other studies collected data at multiple time points, either before, during, and after the intervention conduct, or before, after and at follow-up time-points. Notably, whereas most studies did report the gender of the participants, only six studies reported details about their ethnicities.

The quality of both randomized and quasi-experimental studies was assessed using the Risk of Bias in Non-randomized Studies of Interventions (ROBINS-I) [36, 37]. The full results of the ROBINS-I can be found in Appendix III. For overall risk of bias, only one study was at low risk for bias. Most of the studies were at moderate risk (14 studies) or at serious risk (9 studies) for bias. Looking at the specific domains, risks for bias were found regarding missing data, measurement of outcomes, and selection of reported results. Concerning missing data, the majority (15 studies) had attrition rates lower than 5% and were thus considered to be at low risk. However, about one fifth of the studies had attrition rates higher than 15% and were thus considered to be at high risk of bias due to missing data. Regarding these attrition rates, it is important to note the differences in sample sizes (see Table 4). These sample sizes highlight potential concerns, such as significant attrition in a small sample found within Pidgeon et al.’s (2014) study. Continuing with bias in measurement of outcomes, most studies were at serious risk for bias since there was no attempt to blind the outcome assessors. However, two studies implemented an active control group, which means that the control group also received an intervention as opposed to no intervention. This is an attempt to blind the participants (see Table 4) and the studies were, thus, considered to be only at moderate risk. Regarding the selection of reported results, three studies were at low risk for bias, while the majority had moderate risk due to the absence of preregistrations. Additionally, all included studies were considered to be at low risk for biases related to confounding, participant selection, intervention classification, and deviations from intended interventions. Lastly, we performed a two-sample t-test to address concerns regarding the influence of study quality on effect sizes. Although the mean effect size for studies with a serious risk of bias (mean = 0.7398, n = 16) was higher than that for studies with moderate risk of bias (mean = 0.5849, n = 35), we found no significant difference (t = −0.60828, df = 21.999, p = 0.5492) within our sample, probably due to small sample sizes.

##### Effectiveness of resilience-based interventions

As Table 3 shows, the included interventions used various approaches. For each of these interventions, effect sizes were calculated for their outcomes between baseline and post-test, and between baseline and follow-up. Of the 51 effect sizes, 23 (45%) were significant. The effect sizes of the resilience-directed interventions can be found in Table 5. For resilience-mediated interventions that did not measure resilience, the effect sizes of their primary outcomes can be found in Table 6.

**Table 5:** Effectiveness of Resilience-Directed Interventions

Study	Outcome	M (SD)	Cohen’s D	Significant
Chermack, 2017 [40]	RS	T1: 3.91 (NR) T2: 4.20 (NR)	0.60^a^	Yes
Chitra and Karunanidhi, 2021 [78]	CD-RISC	T1: 59.69 (11.13) T2: 71.77 (12.45) T3^b^: 74.58 (11.37)	T1-2: 1.02 T1-3: 1.32	Yes
Fernandes, 2019 [44]	Teacher Resilience Scale	T1: 3.54 (0.427) T2: 4.24 (0.356)	1.78	Yes
		T1: 3.62 (0.502) T2: 3.93 (0.317)	0.74	Yes
Franco, 2021 [45]	Resiliency Activation	T1: 4.44 (0.13) T2: 4.44 (0.13) T3: 4.46 (0.13)	T1-2: 0 T1-3: 1.23	No
	Resiliency Decompression	T1: 3.48 (0.18) T2: 3.80 (0.18) T3: 4.10 (0.19)	T1-2: 1.78 T1-3: 3.35	Yes
Grabbe, 2019 [46]	CD-RISC10	T1: 29.65 (5.43) T2: 30.42 (3.35) T3: 30.94 (4.07)	T1-2: 0.41^a^ T1-3: 0.39^a^	Yes
Henshall, 2023 [48]	BRS	T1: 3.01 (0.25) T2: 3.02 (0.25)	0.04	No
Hsieh, 2020 [49]	RSA	T1: 153.98 (26.58) T2: 164.15 (23.16)	0.41	Yes
Hsieh, 2020 [49]	RSA	T1: 143.13 (26.28) T2:158.77 (19.20)	0.68	Yes
Im, 2016 [50]	Ego-Resiliency Scale	T1: 44.96 (6.79) T2: 43.92 (6.59)	0.16	No
Janzarik, 2022 [51]	BRS	T1: 3.08 (0.34) T2: 3.12 (0.32)	0.12	No
	CD-RISC	T1: 70.40 (11.84) T2:73.36 (12.38)	0.24	Yes
Joyce, 2019 [52]^c^				
Mache, 2015 [53]	BRCS	T1: 54.3 (17.3) T2: 61.8 (18.4) T3: 61.5 (17.9)	T1-2: 0.42 T1-3: 0.41	Yes
Mao, 2021 [55]	CD-RISC	T1: 57.35 (1.51) T2:69.12 (1.69)	1.026^a^	Yes
Marais, 2016 [57]	RS	T1: 136.54 (12.5) T2: 146.36 (13.95)	0.74	Yes
Mealer, 2014 [58]^c^				
Pidgeon, 2014 [59]^c^				
Slatyer, 2018 [60]^c^				
Spilg, 2022 [63]	CD-RISC	T1: 69.35 (7.71) T3: 73.21 (9.05)	0.46	No
Tonkin, 2018 [64]	CD-RISC10	T1: 3.88 (0.56) T2: 4.04 (0.51)	0.30	No
	EmpRes	T1: 4.15 (0.42) T2: 4.18 (0.47)	0.07	No
Van Der Meulen, 2018 [66]	RS-nl	T1: 4.1 (0.3) T3: 4.1 (0.5)	0	No
	MTQ-48	T1: 177.6 (14.7) T3: 178.3 (15)	0.05	No
Weber, 2019 [67]	RS-13	T1: 4.95 (0.89) T2: 5.14 (1.05)	0.20	No
Wild, 2020 [68]	CD-RISC	T1: 66.49 (14.72) T2: 67.94 (17.01) T3: 68.48 (15.26)	T1-2: 0.09 T1-3: 0.13	No

^a^ reported effect size was calculated by the authors of the study

^b^2-month follow-up

^c^effect size calculation not possible, due to incomplete data

*NR* not reported, *T1* pre-test or baseline measure, *T2* posttest, *T3* 3-month follow-up

**Table 6:** Effectiveness of Resilience-Mediated Interventions

Study	Outcome	M (SD)	Cohen’s D	Significant
Hasani, 2022 [47]	Expanded Nursing Stress Scale	T1: 206 (22.81) T2: 134.3 (30.22) T3^a^: 130.22 (26.70)	T1-2: 2.68 T1-3: 3.05	Yes
Mahaffey, 2021 [54]	Health-Promoting Lifestyle Profile II	T1: 2.69 (0.49) T3: 2.78 (0.50)	0.18	No
	Perceived Stress Scale	T1: 15.05 (7.24) T3: 14.20 (7.14)	0.12	No
	PTSD checklist for DSM-5	T1: 3.64 (8.36) T3: 3.33 (6.72)	0.04	No
	Patient Health Questionnaire	T1: 4.23 (5.54) T3: 3.96 (4.87)	0.05	No
Mistretta, 2018^a^ [77]	Depression (Depression, Anxiety, and Stress Scales (DASS-21))	T1: 4.82 (3.64) T2: 3.64 (3.33) T3: 3.14 (2.90)	T1-2: 0.34 T1-3: 0.51	No
	Stress (DASS-21)	T1: 8.18 (3.74) T2: 5.86 (4.10) T3: 5.82 (3.95)	T1-2: 0.59 T1-3: 0.61	Yes
	Anxiety (DASS-21)	T1: 3.43 (2.11) T2: 2.73 (2.45) T3: 2.18 (1.76)	T1-2: 0.31 T1-3: 0.64	No
	WHO Well-Being Index (WHO-5)	T1: 12.05 (4.51) T2: 14.45 (4.94) T3: 15.36 (4.92)	T1-2: 0.51 T1-3: 0.70	Yes
Mistretta, 2018^b^ [77]	Depression (DASS-21)	T1: 4.43 (3.22) T2: 3.91 (2.66) T3: 3.52 (2.79)	T1-2: 0.18 T1-3: 0.30	No
	Stress (DASS-21)	T1: 7.96 (3.36) T2: 6.43 (2.83) T3: 6.17 (3.02)	T1-2: 0.49 T1-3: 0.56	No
	Anxiety (DASS-21)	T1: 4.43 (3.22) T2: 3.00 (3.18) T3: 2.78 (2.73)	T1-2: 0.45 T1-3: 0.55	No
	WHO-5	T1: 13.09 (3.30) T2:15.78 (2.63) T3: 16.17 (3.24)	T1-2: 0.90 T1-3: 0.94	Yes

^a^Mindfulness-Based Resilience Training

^b^Smartphone Resiliency Training

Looking at the resilience-directed interventions and their outcomes, 29 effect sizes were retrieved, of which 16 (55%) were significant. In general, the effect sizes that were not significant (45%) were small. The one exception was the research by Spilg et al. (2022) who found a medium effect size. Regarding the significant effect sizes, one was small (post-measure), nine effects were medium (56%), with seven at post-measure and two at 3-month measure, and six effects were large (38%), with four at post-measure and two at 3-month measure (see Table 5).

Looking at the resilience-mediated interventions and their outcomes, 22 effect sizes were retrieved, of which 8 (36%) were significant. Most of the effect sizes that were not significant (57%) were small, the other non-significant effect sizes were medium. Regarding the effect sizes that were significant, half was medium (50%) and the other half was large (50%), with two medium and two large effects observed at post-measure, and two medium and two large effects at 3-month measure.

In total, we found 24 significant effect sizes across fourteen separate interventions. Of the eighteen resilience-directed interventions, we found that eleven showed significant improvements in resilience afterwards. Of the corresponding sixteen effect sizes, fifteen were considered medium or large. Combining the effect sizes of the resilience-directed interventions with their intervention strategies, we see some constants for educational workshops and for digital interventions. For educational workshops, interventions with five to 44 sessions yielded medium to large effect sizes. The two exceptions were a one 1-day workshop that included a digital element [46] and one 1-day educational workshop [45]. The remarkable effect sizes of Franco and Christie’s (2021) intervention may be outliers, likely due to small standard deviations. This might be caused by a homogenous sample or limitations within their measurement instrument. A definitive interpretation of this outlier is not possible, due to the lack of statistical analysis. Continuing with digital interventions, most yielded small effect sizes. However, when these digital interventions were combined with other approaches, such as educational workshops or biofeedback, they yielded mixed results with small and medium effect sizes [46, 48–50]. No constants were found for the resilience-mediated interventions.

## Discussion

The aim of this systematic review was twofold: (1) to identify how resilience is defined within different workplace interventions, translated into intervention content, and measured in these interventions; (2) to synthesize the effectiveness of resilience-based interventions in the workplace. We found that most studies use trait-based definitions and measurement instruments to assess the effectiveness of their interventions. Regarding intervention content strategy, group-based educational workshops teaching skills related to resilience or mindfulness were the most popular approaches to impact resilience. While our analysis revealed a majority of studies indicating a beneficial effect of interventions on resilience, we observed that the frequency and duration of educational workshops influenced the magnitude of these effects. However, our findings did not support the notion that any particular intervention type demonstrated superior effectiveness over others.

The persistent usage of trait-oriented definitions of resilience continues, despite criticism regarding its narrow scope [4, 19]. Masten (2007) describes several phases of resilience research, and the most recent phase conceptualizes resilience as a multidimensional process or outcome [10]. The unexpected endurance of the trait perspective in our study indicates a disparity with the evolving nature of resilience research, which is moving beyond static definitions. Another explanation of the frequent use of the trait perspective might lie in incorrect use of the word resilience. As can be seen in Table 3 and 4, some studies (e.g. 55,60,67) seem to use the terms ‘resilience’ and ‘resiliency’ as interchangeable concepts. However, some researchers argue that these two words have different meaning. For example, Prince-Embury [17] mentions that resiliency refers to personal attributes, aligning with trait-oriented definitions, while resilience is an interactive and contextual process incorporating internal and external resources [17]. Solely targeting personal characteristics in an intervention would then make it a resiliency intervention instead of a resilience intervention. This linguistic confusion combined with the discussion regarding the conceptualization of resilience as a trait, process, or outcome raises questions about the accuracy of intervention classifications.

Most studies employed the CD-RISC or RS, which predominantly assess personal characteristics and abilities related to resilience [69–71]. However, these scales, rooted in the trait-orientation, overlook external factors and contextual nuances [19]. Robertson et al. (2015) recommended the use of contextually relevant instruments, but surprisingly, none of the included studies utilized suggested scales like the Resilience at Work Scale [80]. Only three studies adopted context-specific measures [44, 45, 64]. Notably, Tonkin and colleagues (2018) measured both employee resilience and personal resilience and found supporting evidence that employee resilience and personal resilience are, in fact, separate constructs [64]. This evidence supports the recommendation of Robertson et al. (2015) to use more contextually relevant measures. Resilience can differ between domains and contexts; thus, different contexts may require different measurement instruments [4, 23].

A few studies showed inconsistencies in their definitions of resilience, and the way it was measured and translated into intervention content. We found two types of inconsistencies, between: (1) definitions and measurement and (2) content and measurement. Within the first category, we found that studies that choose a trait-oriented definition all choose a measure that fitted this perspective. However, inconsistencies were found within the studies that used a process-oriented or a combined definition. Most inconsistent studies fitted within the second category, with several interventions targeting skills not measured by corresponding scales. This raises concerns about the reliability of measurement tools in accurately capturing the essence of these interventions. This discrepancy might be caused by the increasing popularity of process-oriented definitions of resilience, while the corresponding measurement instruments like the RSA [76] are still in the process of achieving comparable levels of validation and adaptation as their counterparts from the trait-orientation, like the CD-RISC and the RS [69, 72]. Hence, utilization of process-oriented and context-specific measures is lagging behind the well-established trait measures.

In terms of study design and risk of bias, it was remarkable that only a few studies used an active control group and had a preregistration (see Table 4 and Appendix II). Biases may arise depending on the type of control group used in intervention studies. Control groups that receive no intervention or are placed on a waitlist may differ in engagement or expectations compared to the intervention group, potentially affecting outcomes. The use of an active control group—where the control group receives an alternative intervention of similar length and delivery—can help minimize such biases [81]. Although blinding of participants and assessors is an effective way to reduce bias, it is often not feasible in quasi-experimental designs and real-life settings [37, 81]. This challenge is reflected in our findings, where only two of the included studies implemented an active control group. Furthermore, preregistrations can prevent biases within the research and its statistical analyses [82] and can help battle the publication of false findings and selective reporting [83], yet only three of the included studies preregistered their study. Both the implementation of active control groups and use of preregistrations can lower the risk of biases within intervention studies but are only found within a small minority of the included studies. Lastly, in contrary to concerns raised in previous reviews that lower-quality studies may report larger effect sizes, our analysis found no significant difference in mean effect size between categories of risk of bias [84]. However, diagnostic plots indicated greater right-tailed variance in effect sizes for studies with serious risk of bias compared to those with moderate risk, underscoring the importance of cautious interpretation of these findings.

When it comes to reporting participant characteristics, we noticed that whereas most studies report on gender, only a quarter of the studies reported participants’ ethnicity. Both culture and ethnicity can influence resilience; ethnicity relates to shared traits such as origin, language and cultural traditions [85], while culture encompasses beliefs, values and practices shaped by these traits [86]. Within the process of resilience, several reports have shown that ethnicity and culture can serve as a protective factor. Cultural values, for example, can influence one’s beliefs and support system, which can alter one’s coping strategies [86–88]. Additionally, such values might influence supportive relationships, spiritual responses to an adversity, and help make meaning of the situation [8]. Aside from differences in protective factors, different cultures may have varying perceptions of what it means to be resilient [8, 88]. In other words, the way people understand resilience from within their own culture (emic) versus the perspective of an outsider (etic) suggests that resilience and related outcomes might not be a universal construct, and is shaped by cultural traditions [85, 86, 88]. It is notable that many of the included studies do not consider ethnicity and culture, despite evidence suggesting that these factors do impact resilience.

Overall, 11 out of the 18 included studies (approximately 61%) supported the positive impact of resilience-directed interventions. The findings of these studies suggest that resilience-based interventions for employees can have beneficial consequences for both the employees as well as the employers [4, 89]. However, since the studies used different approaches and designs, and the effective interventions have different contents, it is difficult to compare their effectiveness. We only found that within group-based educational workshops, a bigger duration and frequency might yield bigger effect sizes. We, thus, found no evidence that one type of intervention is more effective than others. Results about their effectiveness must thus be interpreted cautiously and within the studies’ own context [19]. Previous literature reviews also found a positive relationship between resilience-based interventions and desirable outcomes, but due to the heterogeneity of the intervention studies in terms of study designs and theoretical backgrounds no definitive conclusions could be drawn about their effectiveness [19, 89].

Although this review sought to examine resilience-based interventions in the workplace broadly, almost all the included studies focused on individual-level strategies, despite acknowledging broader organizational factors such as high workload, staff shortages, and stressful environments as primary causes of stress and burnout (e.g. Hsieh et al. [49]). While several studies briefly mention the role of the organization or leadership, most ultimately shift their focus towards individual interventions, framing them as a feasible or a complementary solution (e.g. Joyce et al. [52], Wild et al. [68]). A few studies included brief reflections on organizational responsibility, without much further development (e.g. Chitra and Karunanidhi [78], Joyce et al. [52], Janzarik et al. [51]), but only two explicitly embraced a socioecological approach [44, 64]. This approach perceives resilience as a multidimensional construct and sociopsychological phenomenon that requires both individual and collective support. It cautions against a hyper-individualistic view and asserts that to be effective, interventions must target both the individual and the environment [44, 64]. This socioecological approach acknowledges the role of external factors for resilience, suggesting that fostering resilience is not solely an individual responsibility, but a shared responsibility that extends to organizations and the work environment [90].

Building on this, resilience is increasingly recognized as a collective, organizational responsibility, rather than solely an individual one [90]. Research highlights the influence of organizational and community factors, such as supportive policies, in shaping resilience, and demonstrates reciprocity whereby investing in individual resilience ultimately strengthens organizational resilience and collective wellbeing [90–92]. The influence of broader contexts underscores the need for more systemic resilience-building approaches [91]. The predominance of individual-level interventions in our findings reveals a gap in the literature, reflecting the need for more research on integrated approaches that address both individual and systemic factors.

This shift in focus from individual to organizational resilience underscores the need for organizational resilience interventions, although this area of research remains in its early stages. Conceptually, studies are aimed at finding the various dimensions of organizational resilience and developing measurement instruments (e.g. [93, 94]), while another review shows that ‘organizational resilience’ also suffers from a proliferation of definitions, with views varying from the adaptation to unexpected events to the development of a healthy workforce [95]. Models like Vercio et al. [96] contribute to progress in the field of resilience interventions by mapping the interplay between individual and organizational resilience, emphasizing vulnerabilities in the employee-organization relationship [96].

## Strengths and Limitations

This study contributes to the field by addressing the persistent jingle-jangle fallacy in resilience research, by systematically categorizing definitions, and evaluating measurement scales. One of its strengths is the use of various synonymous keywords for resilience and interventions, since part of the jingle-jangle fallacy is that some might use a different word but mean the same thing as resilience. Additionally, while our review captures resilience across the public sector—a strength for generalizability compared to reviews focused on single professions—the heterogeneity within the sector presents a potential limitation. Most of the included studies (14 out of 24) focused on healthcare professionals, leaving other public sector disciplines underrepresented. This limits our ability to draw meaningful conclusions about how resilience-based interventions should differ by profession or context, or how they might need to be tailored to specific settings*.* Furthermore, biases were systematically assessed using the ROBINS-I tool, which made it possible to assess both RCT and quasi-experimental studies based on the same criteria. However, one of the criteria regarding the measurement of outcomes might be too strict when applied to public health intervention studies. With this type of studies, it is nearly impossible to fully blind participants for their interventions received and the outcome being measured, especially when it comes to self-reporting questionnaires. Additionally, there were concerns that the COVID-19 pandemic may have influenced the results of some included studies. However, resilience is inherently contextual and often assessed in relation to a stressor, as seen in other studies evaluating interventions during events such as Hurricane Sandy [54] or organizational restructuring [63]. Most of the studies included in this review completed data collection prior to the pandemic, meaning that COVID-19 did not impact these studies. In the case of the RCT conducted during the pandemic [48], randomization ensured that both the control and intervention groups experienced the same context, minimizing bias. However, for the quasi-experimental study, where COVID-19 was only a factor during follow-up [45], this contextual difference could have influenced the results, warranting cautious interpretation. Lastly, most studies seemed to support the positive impact of resilience-based interventions, but this could have been influenced by publication bias. Publication bias occurs when studies with positive outcomes are favoured for publishing, as opposed to studies with negative results [97]. This can distort the interpretation of the true effectiveness of resilience-based interventions.

## Recommendations for Future Research and Practice

Despite the growing popularity of resilience interventions in organizational context, the research field itself is still in an early stage [19, 28]. Our results support the recommendations of Robertson et al. (2015) that future interventions should prioritize alignment of definitions, content, and measurement, ensuring conceptual clarity and consistency. Additionally, researchers should consider the context of their intervention takes in their choice of measurement instrument, aiming for context-specific resilience measures [4]. However, using different measures for each context might only increase our jingle-jangle fallacy, creating a bigger problem. It is crucial to keep a balance and facilitate cross-study comparisons, thus we recommend future researchers to follow the example of Tonkin et al. (2018) and utilize both a general resilience instrument for comparability and a context-specific instrument to assess effectiveness within the context of the intervention itself.

There is, however, a need for more guidelines about which general instrument is best suitable to measure resilience. When researching interventions, there is already the assumption that resilience is a malleable concept. In line with developments in the field of resilience we, thus, recommend that the chosen definitions and measurement scales align with the adaptable nature of resilience [19]. Both the process orientation, with measures such as the RSA [76] and the outcome orientation, where it is measured as an adaptive outcome such as wellbeing, would be more suitable as operationalizations of resilience. Both orientations fit better with the adaptable and dynamic nature of resilience. However, previous reviews made different recommendations about the most suitable orientation for future research (e.g. [4, 15]). Since the outcome orientation leaves room for various outcomes to be measured, it would only hinder comparison. With the current state of knowledge, the process orientation as a basis for definitions and measures might best align with the assumptions of the type of research, while enabling comparison across studies. If the research into resilience-based interventions is to be moved forward, it should leave the narrow trait orientation behind and focus on the broader conceptualizations, such as the process orientation. For the corresponding measures to reach the same level of adaptation and validation as trait measures, more (validation) studies using process and context-specific measures are needed.

Furthermore, future research can help picture how organizational resilience and personal resilience are interconnected. Qualitative studies can explore these two forms of resilience separately while also examining their interplay, identifying shared protective factors and effective strategies for enhancement. This understanding can inform the development of interventions targeting both individual and organizational factors, which is in line with the need to move beyond the predominantly individual-level interventions. Systemic approaches, such as supportive workplace policies, shared visions and inclusive networks that embrace cultural diversity, can cultivate resilience as a joint responsibility, ultimately benefiting both individual wellbeing and overall organizational health [90, 92, 96]. Developing such a holistic perspective will provide a more comprehensive framework for developing resilience-based interventions.

Continuing with intervention content and design, our findings provide tentative evidence that longer duration and higher frequency may lead to larger effect sizes, suggesting a valuable avenue for future research. Exploring the dose–response relationship in resilience-based interventions could help determine whether higher-intensity interventions yield more substantial effects.

Lastly, a greater focus on study design and transparency would help us establish a more reliable basis for the effectiveness of resilience-based interventions. This does not only concern consistency, but also lowering risks of bias by implementing preregistrations and active control groups [81–83, 98, 99]. Additionally, since ethnicity and culture influence the resilience process [85–87], it is important to take this factor into account as well, when studying ethnic heterogeneous populations. Future studies should take these considerations into account when developing a research plan to evaluate resilience-based interventions.

## Conclusion

We found that most studies use trait-based definitions and measures to assess the effectiveness of their resilience-based interventions, and less studies use process- or outcome-oriented definitions and corresponding measures. Most interventions focus solely on individual factors, placing the responsibility for resilience largely on employees themselves, despite evidence that resilience is also shaped by organizational factors. The most popular approach was group-based educational workshops; however, we found no evidence that one type of intervention is more effective than others. Research on resilience-based interventions yields positive effects and the prospect of cultivating a resilient and healthy workforce. Nevertheless, pervasive confusion regarding the effectiveness of these interventions originates from inconsistencies in the theoretical underpinnings and methodological quality across studies. Further advancement in the field calls for a commitment to consistency and transparency in articulating the theoretical foundations, intervention contents, and methodological approaches. This clarity is essential for the development and credibility of resilience-based interventions that promote well-being in the workplace.

## Supplementary Information

Supplementary Material 1.

Supplementary Material 2. [100–104].

Supplementary Material 3.

Supplementary Material 4.

## Data Availability

The datasets generated and/or analysed during the current study are available in the Open Science Framework repository: https://doi.org/10.17605/OSF.IO/SQYXF.

## Abbreviations

ACT: Acceptance-Commitment Therapy
AIT: Attention and Interpretation Therapy
BRCS: Brief Resilience Coping Scale
BRS: Brief Resilience Scale
BT: Biofeedback Training
CBT: Cognitive Behavioral Therapy
CD-RISC: Connor-Davidson Resilience Scale
DASS-21: Depression, Anxiety, and Stress Scales
EmpRes: Employee Resilience Scale
ER-89: Ego-Resiliency Scale
MBRT: Mindfulness-Based Resilience Training
MBSR: Mindfulness-Based Stress Reduction
MTQ-48: Mental Toughness Questionnaire
N/A or N.A.: Not applicable
NR: Not reported
PRISMA: Preferred Reporting for Systematic Reviews and Meta-analysis
PTSD: Post-Traumatic Stress Disorder
QE: Quasi-Experimental
RCT: Randomized Controlled Trial
ROBINS-I: Risk of Bias in Non-randomized Studies of Interventions
RS: Resilience Scale
RSA: Resilience Scale for Adults
SDBT: Smartphone-Deliverd Biofeedback Training
SRS: Short Resilience Survey
SRT: Smartphone Resiliency Training
SST: Sensory Stimulation Therapy
WHO-5: WHO Well-Being Index
