# Supplementary material for: Resilience-based interventions in the public sector workplace: a systematic review
Source: BMC Public Health. 2025 Jan 28;25:350. doi: 10.1186/s12889-024-21177-2 (PMC11773882; doi:10.1186/s12889-024-21177-2)
Supplement: Supplementary file 1 — Supplementary Material 1. [file 12889_2024_21177_MOESM1_ESM.docx]

## Appendix I: Search Queries

### PsycInfo (via OVID)

APA PsycInfo <1806 to April Week 1 2023>

1 "resilience (psychological)".mp. [mp=title, abstract, heading word, table of contents, key concepts, original title, tests & measures, mesh word]

2 (resilien* or hardiness or distress tolerance).mp. [mp=title, abstract, heading word, table of contents, key concepts, original title, tests & measures, mesh word]

3 (intervention* or program* or training* or promotion* or enhancement* or education).mp. [mp=title, abstract, heading word, table of contents, key concepts, original title, tests & measures, mesh word]

4 (employment or employees or personnel or work*).mp. [mp=title, abstract, heading word, table of contents, key concepts, original title, tests & measures, mesh word]

5 exp professional personnel/

6 (randomized controlled trial* or RCT or empirical study or quasi-experiment* or observational study).mp. [mp=title, abstract, heading word, table of contents, key concepts, original title, tests & measures, mesh word]

7 1 or 2

8 4 or 5

9 3 and 7 and 8

10 6 and 9

11 6 and 9

12 6 and 9

13 limit 12 to yr="2013 - 2023"

14 12 and 13

15 limit 12 to English language

16 limit 13 to English language

Number of results: 172

### Web of Science

(((TS=("psychological resilience" OR resilien* OR hardiness OR "distress tolerance")) AND TS=(intervention* OR program* OR training* OR promotion* OR enhancement* OR education*)) AND TS=(employment OR employees OR personnel OR work*)) AND TS=("randomized controlled trial*" OR "RCT" OR "empirical study" OR "quasi-experiment*" OR "observational study" OR "experiment*")

Filter: 2013-01-01 until 2023-04-06

Link to the query: <https://www.webofscience.com/wos/woscc/summary/6227a695-1a99-4c7c-bfee-a7e508e5a25c-7faecc99/relevance/1>

### Pubmed

("psychological resilience" OR resilien* OR hardiness OR "distress tolerance") AND (intervention* OR program* OR training* OR promotion* OR enhancement* OR education) AND (employment OR employees OR personnel OR work*) AND ("randomized controlled trial*" OR RCT OR "empirical study" OR "quasi-experiment*" OR "observational study")

Filter 2013-2023

### Business Source Premier

("psychological resilience" OR resilien* OR hardiness OR "distress tolerance") AND (intervention* OR program* OR training* OR promotion* OR enhancement* OR education) AND (employment OR employees OR personnel OR work*) AND ("randomized controlled trial*" OR RCT OR "empirical study" OR "quasi-experiment*" OR "observational study")

Filter 2013-2023

### Scopus

TITLE-ABS-KEY ( "psychological resilience"  OR  resilien*  OR  hardiness  OR  "distress tolerance" )  AND  TITLE-ABS-KEY ( intervention*  OR  program*  OR  training*  OR  promotion*  OR  enhancement*  OR  education )  AND  TITLE-ABS-KEY ( employment  OR  employees  OR  personnel  OR  work* )  AND  TITLE-ABS-KEY ( "randomized controlled trial*"  OR  rct  OR  "empirical study"  OR  "quasi-experiment*"  OR  "observational study" )  AND  ( LIMIT-TO ( PUBYEAR ,  2023 )  OR  LIMIT-TO ( PUBYEAR ,  2022 )  OR  LIMIT-TO ( PUBYEAR ,  2021 )  OR  LIMIT-TO ( PUBYEAR ,  2020 )  OR  LIMIT-TO ( PUBYEAR ,  2019 )  OR  LIMIT-TO ( PUBYEAR ,  2018 )  OR  LIMIT-TO ( PUBYEAR ,  2017 )  OR  LIMIT-TO ( PUBYEAR ,  2016 )  OR  LIMIT-TO ( PUBYEAR ,  2015 )  OR  LIMIT-TO ( PUBYEAR ,  2014 )  OR  LIMIT-TO ( PUBYEAR ,  2013 ) )
