# Supplementary material for: Resilience-based interventions in the public sector workplace: a systematic review
Source: BMC Public Health. 2025 Jan 28;25:350. doi: 10.1186/s12889-024-21177-2 (PMC11773882; doi:10.1186/s12889-024-21177-2)
Supplement: Supplementary file 2 — Supplementary Material 2. [100–104]. [file 12889_2024_21177_MOESM2_ESM.docx]

## Appendix II: Measurement Instruments

This appendix provides an overview of the thirteen measurement instruments used by the included studies and their theoretical backgrounds (See Table 7).

Table 7: Resilience Measurement Scales and Their Theoretical Backgrounds

| Resilience Scale |  | Resilience Definition | Category | Items and content |
| --- | --- | --- | --- | --- |
| 13-item Resilience Scale (RS-13) | (45) | Resilience connotes the ability to cope effectively when face with adversity, inner strength, optimism, competence, flexibility | Trait | Thirteen items. Shortened version of the RS. German. |
| Brief Resilience Scale | (13) | Resilience is defined as ‘the ability to bounce back from stress’ | Trait | Six items, sees resilience as a unidimensional construct and asks about the most basic meaning of resilience. |
| Brief Resilient Coping Scale | (100) | Resilience as a characteristic approach to situations or stressors and is manifested as cognitive skills, problem-solving ability and attributes that indicate a capacity for action in facing a situation | Trait | Four items, designed to capture tendencies to cope with stress in a highly adaptive manner. For example: looks for creative ways to alter difficult situations. |
| CD-RISC | (40) | Resilience is a measure of one’s stress coping ability and it is defined as one’s personal qualities that enable one to thrive in the face of adversity | Trait | Twenty-five items, divided over five subscales: 1) personal competence, high standards, and tenacity; 2) trust in one’s instinct, tolerance of negative effects, and strengthening effects of stress; 3) positive acceptance of change and secure relationships; 4) control; and 5) spiritual influences. |
| CD-RISC-10 | (41) | Resilience is a measure of one’s stress coping ability and it is defined as one’s personal qualities that enable one to thrive in the face of adversity | Trait | Ten items measuring one factor: resilience |
| Ego-Resiliency Scale (ER-89) | (101) | Resilience is defined as the linkages of the ego structures that keep the personality system within tenable bound or permit the finding again of psychologically tenable adaptational modes. | Trait | Fourteen items, measuring distinct items such as: generosity with friends, quick recovery from being startled, and get over anger reasonably quick |
| Employee Resilience Scale | (72) | Employee capability, facilitated and supported by the organization, to utilize resources to continually adapt and flourish at work, even if/when faced with challenging circumstances | Trait | Nine items focused on employees’ behaviours, such as: effective collaboration while facing challenges, learning from mistakes, and improving way to perform job. |
| Mental Toughness Questionnaire-48 | (64) | n/a |  | Strongly rooted in sports psychology and aimed at mental toughness. Forty-eight items measuring: challenge, commitment, control (emotional and life) and confidence (abilities and interpersonal). |
| Resilience Scale | (43) | Resilience connotes the ability to cope effectively when face with adversity, inner strength, optimism, competence, flexibility | Trait | 25 items, measuring: perseverance, equanimity, self-reliance,  meaningfulness, existential aloneness. |
| Resilience Scale for Adults | (49,102) | Resilience is a multidimensional construct. It refers to important psychological skills and abilities, as well as the ability to use family, social and external support systems to cope better with stress or adversities. | Process | 33 items measuring: positive perceptions of self; positive beliefs and plans for the future; warmth and social competence; structured style in approaching tasks; availability of family support and cohesions; social support and reinforcement from friends and family members |
| Resilience Scale-nl | (46) | Resilience connotes the ability to cope effectively when face with adversity, inner strength, optimism, competence, flexibility | Trait | Dutch version of the RS. Twenty-five items. |
| RS-14 | (44) | Resilience connotes the ability to cope effectively when face with adversity, inner strength, optimism, competence, flexibility | Trait | Shortened version of the RS. Fourteen items. |
| Short Resilience Survey | (103) | Resilience is the ability of an individual, community, or organization to adapt and move on in a positive manner after stressful or adverse situations | Trait | Eight items. Subscales: resilience activation and decompression. |
| Teachers’ Resilience Scale | (104) | Resilience is a form of teacher efficacy and performed two studies to investigate his proposed assets model of resilience. In his proposed model, resilience involves personal strengths, coping skills and social support. | Process | Nine items, aimed at beginning teachers. Assesses how confident teachers feel in dealing with obstacles in school. |
