# Supplementary material for: Resilience-based interventions in the public sector workplace: a systematic review
Source: BMC Public Health. 2025 Jan 28;25:350. doi: 10.1186/s12889-024-21177-2 (PMC11773882; doi:10.1186/s12889-024-21177-2)
Supplement: Supplementary file 3 — Supplementary Material 3. [file 12889_2024_21177_MOESM3_ESM.docx]

## Appendix III: Risk of Bias Assessment - ROBINS-I

| **Study** | **Bias due to confounding** | **Bias in selection of participants into the study** | **Bias in classification of interventions** | **Bias due to deviations from intended interventions** | **Bias due to missing data** | **Bias in measurement of outcomes** | **Bias in selection of the reported result** | **Overall Bias** |
| --- | --- | --- | --- | --- | --- | --- | --- | --- |
| **Chermack, 2017** | Low | Low | Low | Low | Low | Serious | Moderate | Moderate |
| **Chitra, 2021** | Low | Low | Low | Low | Low | Serious | Moderate | Moderate |
| **Fernandes, 2019** | Low | Low | Low | Low | NI | Serious | Moderate | Serious |
| **Franco, 2021** | Low | Low | Low | Low | Moderate | Serious | Moderate | Serious |
| **Grabbe, 2020** | Low | Low | Low | Low | Low | Moderate | Moderate | Moderate |
| **Hasani, 2022** | Low | Low | Low | Low | Low | Serious | Moderate | Moderate |
| **Henshall, 2023** | Low | Low | Low | Low | Low | Serious | Moderate | Moderate |
| **Hsieh, 2020** | Low | Low | Low | Low | Moderate | Serious | Moderate | Serious |
| **Im, 2016** | Low | Low | Low | Low | Low | Serious | Moderate | Moderate |
| **Janzarik, 2022** | Low | Low | Low | Low | Low | Serious | Moderate | Moderate |
| **Joyce, 2019** | Low | Low | Low | Low | Low | Moderate | Low | Low |
| **Mache, 2015** | Low | Low | Low | Low | Low | Serious | Moderate | Moderate |
| **Mahaffey, 2021** | Low | Low | Low | Low | Low | Serious | Moderate | Moderate |
| **Mao, 2021** | Low | Low | Low | Low | Moderate | Serious | Moderate | Serious |
| **Marais, 2016** | Low | Low | Low | Low | Low | Serious | Moderate | Moderate |
| **Mealer, 2014** | Low | Low | Low | Low | Low | Serious | Moderate | Moderate |
| **Mistretta, 2018** | Low | Low | Low | Low | Low | Serious | Moderate | Moderate |
| **Pidgeon, 2014** | Low | Low | Low | Low | Serious | Serious | Moderate | Serious |
| **Slatyer, 2018** | Low | Low | Low | Low | Low | Serious | Moderate | Moderate |
| **Spilg, 2022** | Low | Low | Low | Low | Low | Serious | Low | Moderate |
| **Tonkin, 2018** | Low | Low | Low | Low | Serious | Serious | Moderate | Serious |
| **Van Der Meulen, 2018** | Low | Low | Low | Low | Serious | Serious | Moderate | Serious |
| **Weber, 2019** | Low | Low | Low | Low | Serious | Serious | Moderate | Serious |
| **Wild, 2020** | Low | Low | Low | Low | Serious | Serious | Low | Serious |
